# Integrating stochastic time-dependent travel speed in solution methods for the dynamic dial-a-ride problem

**DOI:** 10.1016/j.ejor.2014.03.005

**Published:** 2014-10-01

**Authors:** M. Schilde, K.F. Doerner, R.F. Hartl

**Affiliations:** a Johannes Kepler University Linz, Institute of Production and Logistics Management, Altenberger Strasse 69, 4040 Linz, Austria; b University of Vienna, Department of Business Administration, Oskar-Morgenstern-Platz 1, 1090 Vienna, Austria

**Keywords:** Dial-a-ride problem, Dynamic stochastic, Time-dependent, Variable neighborhood search, Multiple plan approach, Multiple scenario approach

## Abstract

In urban areas, logistic transportation operations often run into problems because travel speeds change, depending on the current traffic situation. If not accounted for, time-dependent and stochastic travel speeds frequently lead to missed time windows and thus poorer service. Especially in the case of passenger transportation, it often leads to excessive passenger ride times as well. Therefore, time-dependent and stochastic influences on travel speeds are relevant for finding feasible and reliable solutions. This study considers the effect of exploiting statistical information available about historical accidents, using stochastic solution approaches for the dynamic dial-a-ride problem (dynamic DARP). The authors propose two pairs of metaheuristic solution approaches, each consisting of a deterministic method (average time-dependent travel speeds for planning) and its corresponding stochastic version (exploiting stochastic information while planning). The results, using test instances with up to 762 requests based on a real-world road network, show that in certain conditions, exploiting stochastic information about travel speeds leads to significant improvements over deterministic approaches.

## Highlights

• Extensions to four metaheuristics to handle stochastic time-dependent travel speeds.
• Using a state-of-the-art, network-consistent, time-dependent travel time layer.
• A new scheduling algorithm for the dynamic DARP with time-dependent travel speeds.
• Exploiting historical accident information is beneficial in certain conditions.

## 1 Introduction

**Fig. 1:**
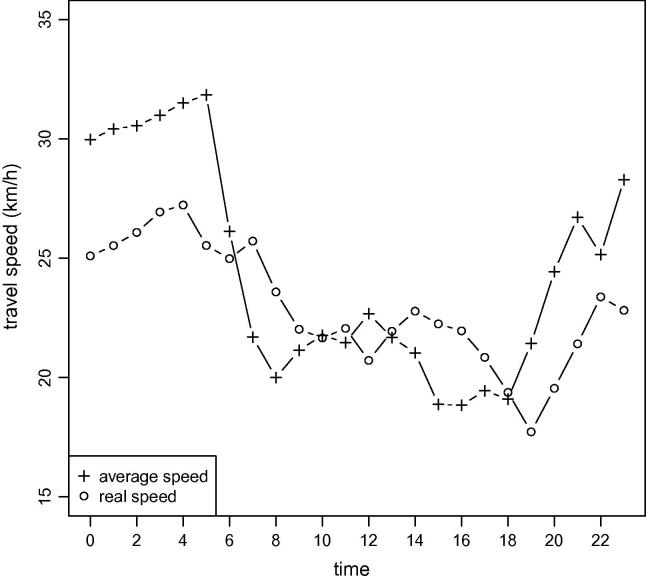
Time-dependent average travel speeds and real (stochastic) travel speeds along a road segment in Vienna over 24 hour.

Most published articles related to vehicle routing problems assume travel speeds that are constant over time (e.g., Muelas, LaTorre, & Peña, 2013; Paquette, Cordeau, Laporte, & Pascoal, 2013; Parragh and Schmid, 2013). In reality, travel speeds rarely are constant but instead depend on factors such as traffic congestion caused by rush hours, accidents, construction sites, or bad weather conditions. An example in Fig. 1 reveals the average travel speeds observed on a specific road segment in the city of Vienna, by time of day. The morning and afternoon peaks (rush hours), which are typical for inner-city roads, are clearly visible. The comparison with the real (stochastic) travel speed observed during one specific day on the same road segment shows that travel speeds are highly sensitive to the time of day, with significant stochastic fluctuations caused by different effects. Therefore, assuming that travel speeds are non-stochastic or even time-independent often causes planned schedules to fail with respect to time windows or ride time limitations.

Some recent publications treat travel speeds as time-dependent, by dividing each day into discrete time intervals, each of which provides a characteristic travel speed for each road within a network (Ehmke, Steinert, & Mattfeld, 2012; Fleischmann, Gietz, & Gnutzmann, 2004; Ichoua, Gendreau, & Potvin, 2003; Kok, Hans, Schutten, & Zijm, 2010; Lorini, Potvin, & Zufferey, 2011; Potvin, Xu, & Benyahia, 2006; Schmid & Doerner, 2010; Xiang, Chu, & Chen, 2008). Even these approaches assume travel speeds to be deterministic though, with the assertion that travel speed, in terms of average values for each interval, is known a priori and not influenced by any stochastic effects. Some authors (Eglese, Maden, & Slater, 2006; Fleischmann, Gnutzmann, & Sandvoß, 2004; Maden, Eglese, & Black, 2010) use a different approach and incorporate time-dependent travel speeds in the process of calculating shortest paths. However, to the best of our knowledge, these algorithms do not take stochastic information about future travel speeds into account to obtain better solutions. Instead, these methods treat travel times as deterministic, so they are restricted to reacting to changes in travel speeds by recalculating the shortest paths.

Travel speeds should be treated as stochastic if we hope to represent reality more precisely. This approach would also improve the reliability and productivity of the planned schedules significantly (Fu, 1999, 2002; Nielsen, Andersen, & Pretolani, 2013; Taş, Dellaert, van Woensel, & de Kok, 2013; Taş, Gendreau, Dellaert, vanWoensel, & de Kok, 2013). In this article, we present variants of stochastic solution methods that use a state-of-the-art, network-consistent, time-dependent travel time layer to take the stochastic influence of future traffic accidents into account while computing vehicle routes. The stochastic accident influence is estimated based on statistical parameters derived from historical, real-world information about accidents with physical injuries collected by different Austrian authorities. There are in fact several factors that can cause stochastic deviations from time-dependent average travel speeds: accidents, temporary construction sites, or large one-time events (e.g., sports games, fares) are just some possible examples. We decided to use data about accidents with physical injuries because of three reasons. First, the respective information is collected systematically by the authorities (and thus available). Second, the influence of major traffic accidents on travel speeds is strong enough to make a difference. Third, traffic accidents occur frequently enough to model them based on statistical information derived from historical data. The numerical results (see Section 5.2) show, that the severity of missed time windows and excess ride times caused by unexpected stochastic changes in travel speed can be reduced significantly by exploiting historical information about traffic accidents with physical injuries.

Especially when conveying passengers, missed time windows and excessive ride times due to changes in travel speeds have a strong negative effect on perceived service quality. This effect becomes even more important when the transported passengers are medical patients or elderly people. The challenge of transporting elderly or disabled people has been widely studied; it is usually modeled as a dial-a-ride-problem (DARP), as introduced in the early 1970s by Wilson and Colvin (1977), Wilson and Weissberg (1976), Wilson, Sussman, Wong, and Higonnet (1971). Healy and Moll (1995) showed that the DARP is NP-hard, and much effort has been dedicated to the development of (meta-)heuristic solution approaches for this problem class, especially in the form of real-world-motivated DARPs (Cordeau & Laporte, 2007; Parragh, Doerner, & Hartl, 2008). A recent review of articles covering the optimization for dynamic ride-sharing was presented by Agatz, Erera, Savelsbergh, and Wang (2012).

In this article, we study the effect of using information about stochastic deviations from time-dependent average travel speeds to plan vehicle routes for a dynamic DARP. This research offers four main contributions:

• We extend four metaheuristic solution approaches to handle stochastic, time-dependent travel speeds in the case of a dynamic DARP.
• We adapt a state-of-the-art, network-consistent, time-dependent travel time layer (i.e., a “method to generate time-dependent travel times that are guaranteed to be network-consistent” (Lecluyse, Sörensen, & Peremans, 2013)) and thereby estimate the stochastic effects of future traffic accidents.
• We propose a new scheduling algorithm for the DARP that is designed specifically to handle time-dependent travel speeds.
• We show that exploiting historical data about traffic accidents using a stochastic planning algorithm has beneficial effects on solution quality in certain conditions.

The remainder of this article is organized as follows. In Section 2, we provide a detailed problem description, followed by an overview of the simulation framework in Section 3 and the solution methods in Section 4. In Sections 5.1 and 5.2, we explain the used test instances and the corresponding computational results, respectively. This article concludes with a summary and short discussion of remaining research questions.

## 2 Problem description

The dynamic DARP with stochastic, time-dependent travel speeds is based on a (directed) real-world road network. Let *d* be the shortest path (with respect to distance, not travel time) between any two nodes in this network. Then T^(t,d)=T^avg(t,d)+T^stoc(t,d) is the time required to travel this path, starting at time *t*, and T^avg(t,d) is the time required to travel the path given the departure time *t*, based on the average vehicle speeds along the path during the affected time intervals. The term T^stoc(t,d) represents the stochastic influence on this travel time, which we assume is revealed the moment a vehicle starts traveling this path. Note that we also assume the shortest paths within the network are constant, not recalculated according to changing traffic situations, but evaluated using the time-dependent or stochastic travel speeds along each path. Although recalculating any single path is not very demanding, doing so for a large number of possible future travel speed scenarios would lead to significant performance problems for an online stochastic solution method. Furthermore, we denote by $\overset{ˇ}{T}(t\text{,}d)$ the time required to travel along this path when the arrival time at the end of the path should be *t*.

Each transportation request *r* consists of two separate nodes $p_{r}\text{,}d_{r}\in N$, representing the pickup and delivery location, respectively. That is, *N* denotes the set of all customer locations in the graph. The time $a_{r}$ represents the time the solution method (i.e., the dispatcher) is informed about transportation request *r* (e.g., by phone). Some requests are static ($a_{r}=0$), but others are dynamic in the sense that they become known only as the day progresses ($a_{r}>0$). For the dynamic DARP, a limited number of vehicles is available to service all requests. We assume that rejecting requests is not permitted (solution feasibility is guaranteed by the soft evening-depot time window, described subsequently).

Each node n∈N is assigned a quantity of $q_{n}=1$ for pickup locations and $q_{n}=-1$ for delivery locations, indicating that one passenger is boarding or leaving the vehicle. The depot node 0 is assigned a quantity of $q_{0}=0$. Each vehicle has a limited capacity of $Q_{\max}=3$, assuming a homogeneous vehicle fleet and homogeneous passengers. Each passenger occupies exactly one seat inside a vehicle, and each vehicle has exactly three seats installed. We do not differentiate between different modes of transportation in this work (interested readers should consult Parragh, Cordeau, Doerner, & Hartl (2012) for a heterogeneous DARP).

Each node *n* (both pickup and delivery) has a time window $\left[e_{n}\text{,}l_{n}\right]$. The depot node 0 has the time window $\left[0\text{,}T_{\max}\right]$, which means that vehicles can leave the depot at any time $e_{0}=0$ or thereafter and should not return later than $l_{0}=T_{\max}$. Here, $T_{\max}$ is the duration of the working shift of the vehicle crew. Arriving at the depot later than $l_{0}$ invokes overtime payments and therefore should be avoided. The beginning and end of each time window is soft. If a service starts before the beginning of the time window, this difference is denoted earliness, and to avoid such earliness, the vehicle can wait before departing to this location or after arrival, just before starting to service this location. However, waiting is only allowed if no patient is currently aboard the vehicle. If service starts after the end of the time window, it is denoted tardiness. A late return to the depot counts as tardiness as well. Therefore, every request can feasibly be inserted into any solution at any time so that incoming requests never have to be rejected.

User inconvenience in terms of excess ride time usually is considered part of the objective function or a constraint on the DARP. In our case, we impose a maximum detour constraint of 30 minutes, in the following sense: Let $d_{\mathrm{direct}}$ be the shortest path from $p_{r}$ to $d_{r}$. Then T^direct(t,ddirect) is the time required to go directly from $p_{r}$ to $d_{r}$, starting at time *t* (based on historical time-dependent average travel speeds). Furthermore, let the time between the planned end of service at $p_{r}$ and the planned start of service at $d_{r}$ be $T_{\mathrm{real}}$. Then the equation Treal⩽T^direct(t,ddirect)+30 should not be violated for any *t*. However, considering the stochastic influence on travel speeds, we cannot guarantee that this equation will strictly hold. If travel time happens to be longer in reality than in the plan, a detour might extend longer than 30 minutes. To penalize this outcome, the amount of time by which a solution violates this maximum detour constraint is denoted as ride time violation.

The top priority when planning the vehicle route is to minimize passenger dissatisfaction. Total costs also must be kept as low as possible. We therefore use a lexicographic objective function:

• The primary objective is to minimize the sum of tardiness, earliness, and ride time violations over all routes.
• The secondary objective is the number of routes (vehicles used).
• The tertiary objective is the total route duration.

In general, solutions are compared against the primary objective. If two solutions are equal in terms of the primary objective, the secondary one is used for comparison. Only if both the primary and the secondary objective are equal for two solutions, does the comparison include the tertiary objective.

## 3 Simulation framework

Algorithm 1 FIFO-approach by Ichoua et al. (2003)

**Table:** 

1: $t\leftarrow t_{0}\text{;}d\leftarrow d_{\mathrm{ij}}\text{;}k\leftarrow \text{GetInterval}(t)$
2: $t^{\prime }\leftarrow t+\frac{d}{v_{\mathrm{ijk}}}$
3: **while**$\left(t^{\prime }>\overset{¯}{t_{k}}\right)$**do**
4: $d\leftarrow d-v_{\mathrm{ijk}}(\overset{¯}{t_{k}}-t)$
5: $t\leftarrow \overset{¯}{t_{k}}$
6: k←k+1
7: $t^{\prime }\leftarrow t+\frac{d}{v_{\mathrm{ijk}}}$
8: **end while**
9: **return** $\left(t^{\prime }-t_{0}\right)$

Because the problem at hand is dynamic, we use a simulation framework developed for the dynamic stochastic DARP with expected return transports (i.e., transports from a hospital back to the patients’ home location are stochastic, while travel speeds are assumed to be time-independent) as the basis for our simulation environment (Schilde, Doerner, & Hartl, 2011). It is designed to keep track of all transportation requests continuously during the execution, and it provides information about incoming new requests to the solution methods whenever necessary. The framework also manages simulation time and travel times. Therefore, after initializing and loading the problem data, the chosen solver module starts, with the simulation time set to zero. Whenever the solver module comes to a point at which it can handle new transportation requests, the simulation framework updates the simulation time according to the actual CPU time elapsed since starting, providing a list of newly known requests to the solver module.

We therefore extended the framework to make it capable of managing stochastic, time-dependent travel speeds (see Section 3.1). We also added an interface that allows the solver modules to request travel times between two locations for a given departure or arrival time. Thus the framework provides travel times, based on historical average speeds within the affected intervals. Only when the simulation time advances are real travel times, including the actual stochastic influences, revealed to the solver modules. The framework accordingly relies on the assumption that true (stochastic) travel speeds along the path to a vehicle’s next stop are revealed to a solver the moment the vehicle departs for this next stop. No update of travel times takes place while traveling the corresponding path. Nor is the actual path recalculated; rather only the corresponding travel speeds along this path are updated at the moment of departure. Additionally, the simulator keeps track of all vehicle departures, which represent the executed solution.

Another essential aspect of this extended framework is that it guarantees the “first-in, first-out” property (FIFO, Ichoua et al. (2003), also known as “non-passing property”, Ahn & Shin (1991)). Two vehicles traversing the same edge in a graph cannot overtake each other. Beyond the logical implications of this property, we enforce it for systemic reasons. Our scheduling algorithm is based on a strategy that requires the calculation of a definite departure time for any given arrival time (see the definition of $\overset{ˇ}{T}$ in Section 2). In such situations, guaranteeing the FIFO property is crucial, because otherwise multiple departure times could result in the same arrival time. We guarantee the FIFO property by using the approach presented by Ichoua et al. (2003), as listed in Algorithm 1. We denote by $t_{0}$ in interval *k* the departure time at location *i*, by $d_{\mathrm{ij}}$ the total distance to be travelled, by $v_{\mathrm{ijk}}$ the velocity on link (i,j) during interval *k*, and by $\overset{¯}{t_{k}}$ the end of interval *k*. The algorithm iteratively determines the distance travelled within each affected interval *k* to obtain a realistic, time-dependent travel time. This approach can easily be inverted, such that the travel time is calculated given a specific arrival time.

We assume that time-dependent average values of travel speeds are known from historical floating car data (FCD). That is, we divide a day into 24 intervals, each implying a specific (known) average travel speed for each road segment in a real-world road network. The corresponding FCD were collected and analyzed during a project in the city of Vienna in 2009. Thus we gain a very realistic representation of the real-world traffic situation during the observation interval, including temporal and geographical correlations between travel speeds on different streets.

Furthermore, we assume historical accident information is available. For this purpose, we use accident information collected partly by Statistics Austria and partly by the Automated Data Processing, Information and Communications Technology department at the City of Vienna (Municipal Department 14). This includes the geographical location of 4078 traffic accidents that resulted in personal injury within the city limits of Vienna during 2011 (information about accidents without personal injuries is not collected). With this data set, we determined the probability of a severe accident within the boundaries of each of the 23 Viennese districts during each hour of the day.

### 3.1 Congestion circles

To determine the influence of traffic accidents on actual travel speeds, we use the concept of a network-consistent, time-dependent travel time layer, as proposed by Lecluyse et al. (2013). The main idea is to model traffic accidents as congestion circles. An accident’s influence on traffic is thus determined by a factor representing accident severity and the area covered by a circle centered at the accident location. The size of this circle expands up to its maximum diameter, stagnates for some time, and then slowly shrinks back to size 0. The actual influence on travel speeds on any link in the underlying graph can be calculated on the basis of the severity factor of the accident, weighted by the percentage of the link currently covered by the circle area. For brevity, we refer interested readers to Lecluyse et al. (2013) for the calculation details.

The exact calculation of the timing information (e.g., when a vehicle on a given link passes the time-dependent circle boundary, when it leaves the circle again) is computationally quite demanding and needs to be performed online for each circle and each link inside the network. Therefore, we use an approximation to allow for real-time calculation within the solution framework. Specifically, we do not superimpose the travel time layer on the actual road network but use bee-line connections between the origin and destination of the vehicle instead.

For each origin–destination pair and each travel speed interval, we determine the maximum percentage of the bee-line section covered by the congestion circle. Next, we weight this factor by the percentage of the respective interval duration during which the link is actually covered by the circle (taking into account the time when the circle does not cover the link at all, the time until the circle reaches its maximum radius, the stagnation time at full extension, the time during which the circle shrinks, and the time when the circle does not cover the link any more). The resulting factor then can reduce the actual travel speed on the given path during the respective interval.

Using this method, we determine the expected influence of all accidents known from historical data on each origin–destination pair. This expected influence provides the basis for evaluating the proposed methods (i.e., resulting travel speeds are revealed in the solution methods as real travel speeds). Thus we ensure that all solution methods are evaluated using real-world accident information.

**Fig. 2:**
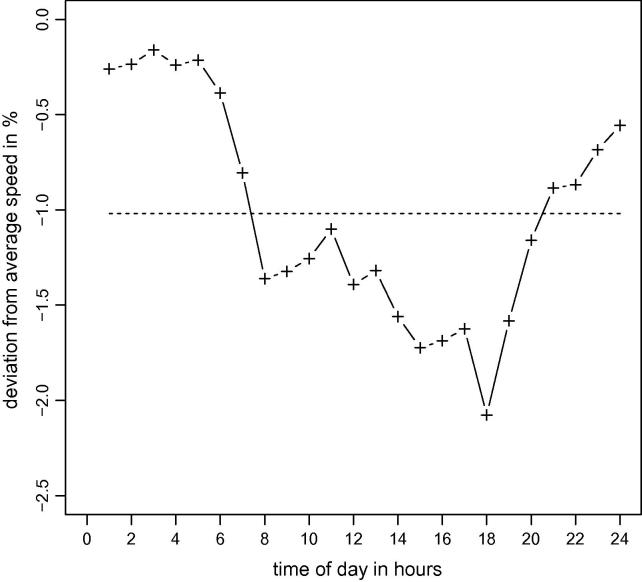
Expected average accident influence on all links during the 24 intervals of a day (e.g., −1% indicates that all accidents that happening during a year are expected to cause an average decrease in travel speed of one percent on all links in the network). The dashed line represents the average over all intervals.

A graphical representation of the expected accident influence as an average over all links within a single test instance appears in Fig. 2. The morning and evening rush hour clearly arise; the higher traffic densities during these periods imply a higher likelihood of traffic accidents. The overall average influence of accidents on any link inside this test instance is a 1.02% reduction in travel speed (dashed line in Fig. 2). The maximum influence on any link in any interval in this instance is a 4.87% reduction in travel speed.

## 4 Solution methods

Our solution approaches proceed as follows: First, the sequence of transportation requests inside each route is determined. Second, the feasibility and timing for each route is determined using a scheduling algorithm. For the sequencing, we first generate an initial solution (see Section 4.2), which can be improved using one of four metaheuristic solution methods, as described in Sections 4.3, 4.4 and 4.5. For the second phase, we present a new alternative scheduling algorithm for the DARP that is designed especially to handle time-dependent travel speeds. This method, the block scheduling algorithm (BSA), is described next.

### 4.1 Block scheduling algorithm

The block scheduling algorithm is inspired by two existing ideas: the concept of scheduling blocks presented by Jaw, Odoni, Psaraftis, and Wilson (1986) (which was also used by Fu (2002) and is similar to the concept of zero split points presented by Parragh, Doerner, & Hartl (2010)) and the forward time slack concept proposed by Savelsbergh (1992). A service block is a sequence of nodes within a route that starts and ends with the vehicle being empty. The forward time slack is originally defined as the maximum amount of time the departure from a node can be delayed without causing the route to be infeasible. For our scheduling algorithm, we define the forward time slack as the maximum amount of time the start of service at a node can be delayed without increasing tardiness with respect to its time window.

Instead of adapting one of the two most commonly used scheduling algorithms proposed for the deterministic DARP (Cordeau & Laporte, 2003; Hunsaker & Savelsbergh, 2002) to the requirements of the dynamic DARP with stochastic, time-dependent travel speeds, we decided to introduce a completely new scheduling algorithm, the BSA. Changes to the two existing algorithms, which are required for several reasons, would be too extensive. In particular, shifting services with respect to the time they are performed can have a direct influence on all subsequent travel times (i.e., shifting it into the future causes future vehicle movement to be shifted into intervals with different travel speeds, thus changing the time required for these movements). The problem at hand also includes only soft time windows, soft maximum ride time constraints (due to the stochastic influences on travel speeds), and no waiting time allowed whenever a person is aboard a vehicle.

The major difference between the two existing scheduling algorithms and our BSA is that the existing methods are based on the assumption that delaying the departure to a stop along the route, within the limits represented by the forward time slack, does not have a negative upstream effect on consecutive stops’ timing. In the case of time-dependent travel speed, this assumption is not valid though. Therefore, our BSA is designed to handle the effects caused by time-dependent travel speeds. It includes an iterative correction phase, compensating for such effects automatically.

Our description of the BSA depends on the assumption that all information related to solution quality (i.e., tardiness, earliness, ride time violations, number of vehicles used, and total route duration) is calculated a posteriori in an additional evaluation step after the scheduling algorithm. This information does not have any influence on the scheduling of a given vehicle route but would add further complexity to the presented algorithm outlines. Nevertheless, including the required calculations in an actual implementation of the BSA is possible.

Algorithm 2 Block scheduling algorithm outline

**Table:** 

1:	// STAGE 1
2:	**for** all stops along the route **do**
3:	Determine timing without waiting time
4:	**if** vehicle is empty before this stop **then**
5:	Add waiting time before this stop if needed
6:	Remember new block characteristics
7:	**else**
8:	Update block characteristics
9:	**end if**
10:	**end for**
11:	// STAGE 2
12:	**for** all blocks in backwards order **do**
13:	**if** block can and should be shifted **then**
14:	Shift block by adding waiting time in front
15:	Update block characteristics
16:	**while** shift caused critical problems **do**
17:	Undo part of the shift to correct problems
18:	Update block characteristics
19:	**end while**
20:	**end if**
21:	**end for**

**Fig. 3:**
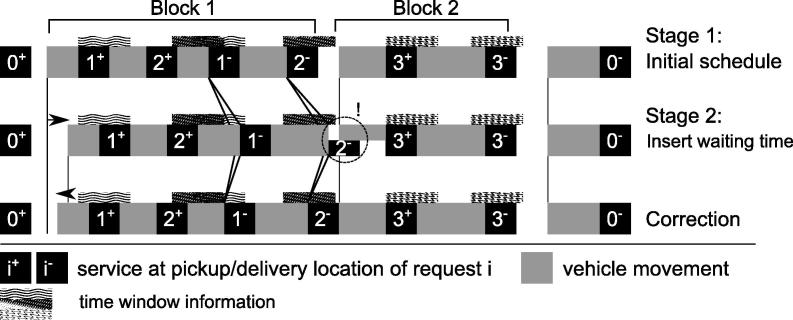
Example BSA. The first stage creates an initial schedule. The second stage inserts additional waiting time before the first block to reduce earliness when arriving at the pickup node for the second request. Due to changes in travel speed, the travel time between locations $2^{+}$ and $1^{-}$ as well as between $1^{-}$ and $2^{-}$ increases, causing the block to overlap the next block, which must be corrected.

The BSA is a two-stage method, as outlined in Algorithm 2; we provide a simple example of how the BSA works in Fig. 3. In the first stage, the algorithm creates an initial schedule for the given route by using forward propagation (Lines 2–10). Waiting times are permitted only directly before departing for a pickup if the vehicle is empty (not after arriving at the pickup location). Such a point along the route at which the vehicle is empty also indicates the beginning (and end) of a service block. During this first stage, all the required parameters for the respective service blocks are stored for the second stage. This initial schedule then gets refined in the second stage with the introduction of additional waiting time before the service blocks, to reduce earliness with respect to the time windows (Lines 12–21). This second stage moves through the found blocks in backward order and introduces just enough waiting time prior to the first stop of each block to minimize earliness without increasing tardiness. The time-dependent nature of travel speeds means that this shift can cause problems if the changing travel times cause the current block to overlap the next block. However, the effect can be addressed by iteratively removing part of the inserted waiting time again.

Algorithm 3 Block scheduling algorithm: Stage 1

**Table:** 

1:	$i\leftarrow 0\text{;}Q_{0}\leftarrow 0\text{;}\Theta \leftarrow \emptyset$;
2:	**for**$n=1\text{,}\ldots \text{,}|S|:D_{n-1}>R$**do**
3:	**if**$Q_{n-1}+q_{n}>Q_{\max}$**then**
4:	Infeasible
5:	**end if**
6:	Bn←Dn-1+T^(Dn-1,dn-1,n)
7:	$D_{n}\leftarrow B_{n}+p_{n}$
8:	**if**$Q_{n}=0$**then**
9:	**if**$B_{n}<e_{n}$**then**
10:	$B_{n}\leftarrow e_{n}$
11:	$D_{n-1}\leftarrow B_{n}-\overset{ˇ}{T}(B_{n}\text{,}d_{n-1\text{,}n})$
12:	$D_{n}\leftarrow B_{n}+p_{n}$
13:	**end if**
14:	i←i+1;Θ←Θ∪{i}
15:	$\Theta_{i}^{\mathrm{start}}\leftarrow n$
16:	$\Theta_{i}^{\mathrm{waiting}}\leftarrow D_{n-1}-B_{n-1}-p_{n-1}$
17:	$\Theta_{i}^{\mathrm{earliness}}\leftarrow \max\{e_{n}-B_{n}\text{,}0\}$
18:	$\Theta_{i}^{\mathrm{slack}}\leftarrow \max\{l_{n}-B_{n}\text{,}0\}$
19:	**else**
20:	$\Theta_{i}^{\mathrm{earliness}}\leftarrow \max\{\max\{e_{n}-B_{n}\text{,}0\}\text{,}\Theta_{i}^{\mathrm{earliness}}\}$
21:	$\Theta_{i}^{\mathrm{slack}}\leftarrow \min\{\max\{l_{n}-B_{n}\text{,}0\}\text{,}\Theta_{i}^{\mathrm{slack}}\}$
22:	**end if**
23:	$Q_{n}\leftarrow Q_{n-1}+q_{n}$
24:	**end for**

Algorithm 4 Block scheduling algorithm: Stage 2

**Table:** 

1:	**for**$i=|\Theta |-1\text{,}\ldots \text{,}1:D_{\Theta_{i}^{\mathrm{start}}-1}>R$**do**
2:	**if**$\left(\Theta_{i}^{\mathrm{slack}}>0)\wedge (\Theta_{i}^{\mathrm{earliness}}>0)\wedge (\Theta_{i+1}^{\mathrm{waiting}}>0\right)$**then**
3:	$s\leftarrow \min\{\Theta_{i}^{\mathrm{slack}}\text{,}\Theta_{i}^{\mathrm{earliness}}\text{,}\Theta_{i+1}^{\mathrm{waiting}}\}$
4:	$B_{\mathrm{orig}}\leftarrow B_{\Theta_{i+1}^{\mathrm{start}}-1}$
5:	$\Theta_{i}^{\mathrm{waiting}}\leftarrow \Theta_{i}^{\mathrm{waiting}}+s$
6:	$B_{\Theta_{i}^{\mathrm{start}}}\leftarrow B_{\Theta_{i}^{\mathrm{start}}}+s$
7:	$D_{\Theta_{i}^{\mathrm{start}}-1}\leftarrow B_{\Theta_{i}^{\mathrm{start}}}-\overset{ˇ}{T}(B_{\Theta_{i}^{\mathrm{start}}}\text{,}d_{\Theta_{i}^{\mathrm{start}}-1\text{,}\Theta_{i}^{\mathrm{start}}})$
8:	$\Theta_{i}^{\mathrm{tardiness}}\leftarrow 0$
9:	ϕ←0
10:	**for**$n=\Theta_{i}^{\mathrm{start}}+1\text{,}\ldots \text{,}\Theta_{i+1}^{\mathrm{start}}-1$**do**
11:	$D_{n-1}\leftarrow B_{n-1}+p_{n-1}$
12:	Bn←Dn-1+T^(Dn-1,dn-1,n)
13:	$\Theta_{i}^{\mathrm{tardiness}}\leftarrow \max\{\max\{B_{n}-l_{n}\text{,}0\}\text{,}\Theta_{i}^{\mathrm{tardiness}}\}$
14:	$\Theta_{i}^{\mathrm{slack}}\leftarrow \min\{\max\{l_{n}-B_{n}\text{,}0\}\text{,}\Theta_{i}^{\mathrm{slack}}\}$
15:	**if**$n=\Theta_{i+1}^{\mathrm{start}}-1$**then**
16:	$\Theta_{i+1}^{\mathrm{waiting}}\leftarrow D_{n}-B_{n}-p_{n}$
17:	**if**$\left((\phi <\phi_{\max})\wedge (\Theta_{i}^{\mathrm{tardiness}}>0))\vee (\Theta_{i+1}^{\mathrm{waiting}}<0\right)$**then**
18:	ϕ←ϕ+1
19:	$s_{\mathrm{corr}}\leftarrow ((B_{n}-B_{\mathrm{orig}})/s)|\min\{-\Theta_{i}^{\mathrm{tardiness}}\text{,}\Theta_{i+1}^{\mathrm{waiting}}\}|$
20:	$s\leftarrow s-s_{\mathrm{corr}}$
21:	$\Theta_{i}^{\mathrm{waiting}}\leftarrow \Theta_{i}^{\mathrm{waiting}}-s_{\mathrm{corr}}$
22:	$B_{\Theta_{i}^{\mathrm{start}}}\leftarrow B_{\Theta_{i}^{\mathrm{start}}}-s_{\mathrm{corr}}$
23:	$D_{\Theta_{i}^{\mathrm{start}}-1}\leftarrow B_{\Theta_{i}^{\mathrm{start}}}-\overset{ˇ}{T}(B_{\Theta_{i}^{\mathrm{start}}}\text{,}d_{\Theta_{i}^{\mathrm{start}}-1\text{,}\Theta_{i}^{\mathrm{start}}})$
24:	$n\leftarrow \Theta_{i}^{\mathrm{start}}+1$
25:	$\Theta_{i}^{\mathrm{tardiness}}\leftarrow 0$
26:	**end if**
27:	**end if**
28:	**end for**
29:	**end if**
30:	**end for**

A detailed outline of the two stages appears in Algorithms 3 and 4. We rely on the following notation: *S* is the set of nodes to be visited by the current route (in chronological order, 0 and |S| are the indices of the depot nodes), $Q_{n}$ is the number of passengers aboard the vehicle when it arrives at node $n\in S\text{,}B_{n}$ is the beginning time of service at node $n\text{,}D_{n}$ is the departure time from node *n*, $p_{n}$ is the service time at node $n\text{,}\Theta_{i}$ is used to store information about service block i,R denotes currently simulated point in time, and $d_{n-1\text{,}n}$ denotes the shortest path between two consecutive stops.

The first stage consists of creating an initial schedule using forward propagation (Algorithm 3). Waiting times (to reduce earliness with respect to time windows) are only permitted if no person is aboard the vehicle (Lines 10–12). Because all time windows and the maximum ride time limitation are soft, the only definitive infeasibility would be a violation of the vehicle capacity (Line 4). Thus we obtain a feasible schedule that consists of a sequence of service blocks, connected by waiting time and subsequent dead-heading movement. During this first phase, we keep track of four properties for each block *i* (Lines 15–21): the starting index ($\Theta_{i}^{\mathrm{start}}$), the maximum earliness within the block ($\Theta_{i}^{\mathrm{earliness}}$), the minimum forward time slack within the block ($\Theta_{i}^{\mathrm{slack}}$) and the waiting time before the block ($\Theta_{i}^{\mathrm{waiting}}$).

In the second stage, the algorithm steps backward through the schedule blocks and even postpones entire blocks to reduce earliness (Algorithm 4). Thus the waiting time before each block *i* is increased by the minimum of $\Theta_{i}^{\mathrm{earliness}}$, $\Theta_{i}^{\mathrm{slack}}$, and $\Theta_{i+1}^{\mathrm{waiting}}$ (Lines 3–7). All timings inside the block and the block’s properties get updated accordingly, using forward propagation again (Lines 11–14). During this process, the algorithm keeps track of the maximum tardiness within each block ($\Theta_{i}^{\mathrm{tardiness}}$). Thus we obtain a schedule that minimizes earliness and tardiness with respect to time windows without waiting time within a service block. However, because of the time-dependent nature of travel speeds, postponing a service block can cause the block to overlap the next block (i.e., we would need to shift the subsequent block, which we already shifted in a previous iteration) or an increase in tardiness within the block. Vehicle movements might be shifted into later time intervals with lower travel speeds. The BSA iteratively compensates for this effect before continuing with the preceding block by determining the amount by which the block overlaps the subsequent block caused by the current block movement relative to the performed postponement. Then it undoes part of the block’s postponement accordingly (Lines 19–25). At this point, either tardiness inside the current block or overlapping the next block have been induced by the shifting operation (or, if a correction was already performed, by what remains of the original shift). Therefore, the performed shift is partly undone by shifting the block backward in time by $s_{\mathrm{corr}}=((B_{n}-B_{\mathrm{orig}})/s)|\min\{-\Theta_{i}^{\mathrm{tardiness}}\text{,}\Theta_{i+1}^{\mathrm{waiting}}\}|$. The term $(B_{n}-B_{\mathrm{orig}})/s$ represents a factor that indicates by how much the end of this block shifts forward in time if the beginning of the block is shifted forward by one time unit. Using this factor (which is an approximation, because the exact effect depends on the actual position of the block with respect to the time intervals), the amount of tardiness or overlapping that needs to be corrected can be weighted. This correction is performed iteratively, as long as the block overlaps the subsequent block. If the correction is performed solely to compensate for an increase in tardiness inside the block caused by the shift, it is executed only up to $\phi_{\max}$ times. This limitation helps to avoid excessive computation time. Overlapping of the subsequent block must be eliminated completely before continuing.

### 4.2 Initial solution

Algorithm 5 Modified cheapest insertion heuristic

**Table:** 

1:	R← ListOfKnownRequests ()
2:	x← AddEmptyRoute ()
3:	**for**r∈R**do**
4:	**if**$\Delta (x\text{,}r)>\Delta (x^{+}\text{,}r)$ AND V(x)>0**then**
5:	x← InsertRequestIntoNewRoute (x,r)
6:	**else**
7:	x← InsertRequestAtBestPosition (x,r)
8:	**end if**
9:	**end for**
10:	**return** *x*

To create an initial solution for all our algorithms, we used an adapted version of the cheapest insertion heuristic proposed by Rosenkrantz, Stearns, and Lewis (1974) for the traveling salesperson problem, as outlined in Algorithm 5. We denote by Δ(x,r) the decrease in solution quality with respect to the objective function caused by inserting a request *r* into an existing route in solution *x* and by $\Delta (x^{+}\text{,}r)$ the decrease caused by inserting *r* into a new route in solution *x*. Furthermore, V(x) represents the number of vehicles still unused in a given (partial) solution *x*.

### 4.3 Dynamic variable neighborhood search

The first solution method we adapt to the requirements of our problem is a dynamic variable neighborhood search (VNS), presented for the DSDARP by Schilde et al. (2011). Algorithm 6 provides the outline. The main difference with traditional VNS (Hansen & Mladenović, 2005; Mladenović & Hansen, 1997) appears in Line 5: Because the problem at hand is dynamic, the algorithm must ensure that dynamically arising requests get taken into account during execution. Therefore, the method inserts such requests into the current solution at the beginning of each iteration (i.e., before performing the shaking step). The iterative search process stops when it reaches the end of the simulated working shift and all transportation requests have been completely serviced (a late return to the depot is possible).

Algorithm 6 Dynamic VNS

**Table:** 

1:	x← InitialSolution ()
2:	N(κ)← SelectFirstNeighborhood ()
3:	**while** StoppingCriterionNotMet () **do**
4:	x← RescheduleWithTrueTravelSpeeds (*x*)
5:	x← InsertNewRequests (*x*)
6:	$x^{\prime }\leftarrow$ ShakeSolution (x,N(κ))
7:	x← MoveOrNot ($x\text{,}x^{\prime }$)
8:	N(κ)← SelectNextNeighborhood (κ)
9:	**end while**
10:	**return** *x* as best found solution

Whenever a vehicle departs for the next stop along its route, the real travel time is revealed to the solution method by the simulation framework. Thus, the algorithm reschedules all subsequent requests in this route (Line 4). The remainder of the dynamic VNS corresponds to the reduced VNS concept reported by Hansen and Mladenović (2005), so we do not use an additional local search step after the shaking step. Extensive testing has shown that the neighborhood operators implicitly include some local search behavior, because reinsertion into the same route is allowed, which makes an additional local search step relatively ineffective.

As in the previously reported version of dynamic VNS (Schilde et al., 2011), we use a set of four neighborhood operators, based on those reported by Parragh et al. (2010), during the shaking phase of the dynamic VNS algorithm (Line 6). Each operator applies up to five different intensity levels κ={1…5}. The first operator randomly removes κ transportation requests from a randomly selected route and reinserts them into any route at the position where they fit best, such that it causes the smallest possible deterioration in solution quality. The second operator randomly selects two routes and removes up to κ consecutive requests from each of them, starting at a randomly selected position. The removed requests then can be reinserted into the corresponding other route at the position where they fit best. The third operator starts by randomly selecting an origin route and a destination route, then removes a sequence of up to κ consecutive requests from the origin route and reinserts them into the destination route where they fit best. It iterates κ times, using the destination route as the new origin route and a randomly selected new destination route. Finally, the fourth operator randomly selects one route and determines all positions inside the route at which the corresponding vehicle is empty (“zero split points”). It then randomly selects two of these points, whereby up to κ-1 other such points may be in between these two points. After removing all transportation requests between the selected points, it reinserts these requests into any route at the position where they fit best.

We start our search using the first operator with intensity level κ=1. During each iteration we randomly create one neighboring solution. If this solution is better than the current incumbent solution, it replaces the current incumbent, and the next iteration continues with the first neighborhood operator with the lowest intensity level (κ=1). Otherwise, the intensity level increases for the next iteration (κ=κ+1). If the maximum value for the intensity is reached, the next neighborhood operator with intensity level κ=1 is used (sequence: move → swap → chain → zero split). If the maximum intensity occurs with the last neighborhood operator, the first operator with the lowest intensity is used again (Line 8).

### 4.4 Dynamic stochastic variable neighborhood search

The second algorithm we apply to our problem is an adaptation of the dynamic stochastic variable neighborhood search approach (dynamic S-VNS), which is based on the dynamic S-VNS algorithm presented for the DSDARP (Schilde et al., 2011). The primary goal of the S-VNS concept is to determine the quality of any given solution using samples of future developments. Its outline is in Algorithm 7.

Algorithm 7 Dynamic S-VNS

**Table:** 

1:	$x^{*}\leftarrow x\leftarrow$ InitialSolution (); $\overset{‾}{Z}\leftarrow 1$
2:	N(κ)← SelectFirstNeighborhood ()
3:	**while** StoppingCriterionNotMet () **do**
4:	x← RescheduleWithTrueTravelSpeeds (*x*)
5:	$x^{*}\leftarrow$ RescheduleWithTrueTravelSpeeds ($x^{*}$)
6:	**if** NewRequestsPresent () **then**
7:	$x^{*}\leftarrow$ InsertNewRequests ($x^{*}$)
8:	$x\leftarrow x^{*}$
9:	**end if**
10:	$x^{\prime }\leftarrow$ ShakeSolution (x,N(κ),Z)
11:	Z← SelectSampledAccidents ($\overset{‾}{Z}$)
12:	x← MoveOrNot ($x\text{,}x^{\prime }\text{,}Z$)
13:	$x^{*}\leftarrow$ MoveOrNot ($x^{*}\text{,}x^{\prime }\text{,}Z$)
14:	N(κ)← SelectNextNeighborhood (κ)
15:	$\overset{‾}{Z}\leftarrow$ AdaptSampleSize ($\overset{‾}{Z}$)
16:	**end while**
17:	**return** $x^{*}$ as best found solution

Dynamic S-VNS takes stochastic information about future travel speeds into consideration while planning and evaluates solutions on the basis of samples of future travel speeds. Therefore, we would use a set of sampled accident scenarios, created in a preprocessing step. These samples of possible future traffic accidents are created according to the distribution parameters determined from historical data, including the temporal and spatial distribution. A dynamically selected subset of $\overset{‾}{Z}$ of these samples in combination with the historical time-dependent average travel speeds is then used by the stochastic method to calculate the respective travel times for each vehicle movement, using the procedure described in Section 3.1.

The overall structure of dynamic S-VNS is similar to that for dynamic VNS. At the beginning of each iteration, the algorithm reschedules the current incumbent solution and the best-so-far solution using real travel speeds if they are known by now (Lines 4 and 5). If new requests have become known during the last iteration, they enter the best-so-far solution (Line 7), and the search process continues. The subsequent shaking step uses the same neighborhood structures described for dynamic VNS (Line 10). If the new solution has a better average solution quality with respect to the current set of samples, it is selected as the new current incumbent solution (Line 12). Also, the best-so-far solution gets updated on the basis of the same subset of sampled accidents (Line 13). Because the computational complexity of calculating future travel speeds depends on the size of the subset of sampled accidents $\overset{‾}{Z}$, this number should be rather small. Our algorithm starts using a single sample ($\overset{‾}{Z}=1$) and adapts the number of samples dynamically, depending on the execution time of the solution evaluation. If the evaluation takes less than 1 second, $\overset{‾}{Z}$ increases by 1; otherwise, it decreases by 1 (Line 15).

### 4.5 Multiple plan approach and multiple scenario approach

Algorithm 8 Structure of the Multiple Plan Approach

**Table:** 

1:	x← InitialSolution (); P←{x}
2:	N(κ)← SelectFirstNeighborhood ()
3:	**while** StoppingCriterionNotMet () **do**
4:	$\overset{¯}{x}\leftarrow$ RescheduleWithTrueTravelSpeeds ($\overset{¯}{x}$) $\forall \overset{¯}{x}\in P$
5:	**for**$\overset{¯}{x}\in P$
6:	**if**$\left(\overset{¯}{x}\;\neq \;x)\wedge (\text{Timeout}(\overset{¯}{x}\text{,}x)\vee \text{Departure}(\overset{¯}{x}\text{,}x)\right)$**then**
7:	$P\leftarrow P⧹\{\overset{¯}{x}\}$
8:	**else if** NewRequestsPresent () **then**
9:	InsertNewRequests ($\overset{¯}{x}$)
10:	**end if**
11:	**end for**
12:	**if** HasChanged (*P*) **then**
13:	x← SelectCurrentIncumbent (*P*)
14:	**end if**
15:	$x^{\prime }\leftarrow$ ShakeSolution (x,N(κ))
16:	**if**$x^{\prime }\;\notin \;P$**then**
17:	$P\leftarrow P\cup \{x^{\prime }\}$
18:	**end if**
19:	N(κ)← SelectNextNeighborhood (κ)
20:	**end while**

Algorithm 9 Structure of the Multiple Scenario Approach

**Table:** 

1:	x← InitialSolution (); $P\leftarrow \{x\}\text{;}\overset{‾}{Z}\leftarrow 1$
2:	N(κ)← SelectFirstNeighborhood ()
3:	**while** StoppingCriterionNotMet () **do**
4:	$\overset{¯}{x}\leftarrow$ RescheduleWithTrueTravelSpeeds ($\overset{¯}{x}\text{,}Z$) $\forall \overset{¯}{x}\in P$
5:	**for**$\overset{¯}{x}\in P$**do**
6:	**if**$\left(\overset{¯}{x}\;\neq \;x)\wedge (\text{Timeout}(\overset{¯}{x}\text{,}x)\vee \text{Departure}(\overset{¯}{x}\text{,}x)\right)$**then**
7:	$P\leftarrow P⧹\{\overset{¯}{x}\}$
8:	**else if** NewRequestsPresent () **then**
9:	InsertNewRequests ($\overset{¯}{x}$)
10:	**end if**
11:	**end for**
12:	**if** HasChanged (*P*) **then**
13:	Z← SelectSampledAccidents ($\overset{‾}{Z}$)
14:	x← SelectCurrentIncumbent (P,Z)
15:	$\overset{‾}{Z}\leftarrow$ AdaptSampleSize ($\overset{‾}{Z}$)
16:	**end if**
17:	$x^{\prime }\leftarrow$ ShakeSolution (x,N(κ))
18:	**if**$x^{\prime }\;\notin \;P$**then**
19:	$P\leftarrow P\cup \{x^{\prime }\}$
20:	**end if**
21:	N(κ)← SelectNextNeighborhood (κ)
22:	**end while**

With dynamic VNS and dynamic S-VNS, we directly compare a purely deterministic approach (dynamic VNS) with a stochastic approach (dynamic S-VNS). To check if the observed differences can be expected as a general outcome when comparing deterministic and stochastic methods for this problem, we implement an additional pair of methods. To allow for direct comparisons between the two additional algorithms, we again use two very similar concepts. Namely, we adapt the multiple plan approach (MPA, see Algorithm 8) and the multiple scenario approach (MSA, see Algorithm 9) to the requirements of the problem at hand. Both methods were originally proposed for the dynamic vehicle routing problem with time windows and stochastic customers by Bent and Van Hentenryck (2004).

We base both methods on the adaptations for the DSDARP (Schilde et al., 2011) and introduce all modifications required to cope with stochastic, time-dependent travel speeds. As an underlying search procedure, we use our implementation of the dynamic VNS. Thus we can guarantee that all differences found between the results of our four methods are caused by the essential conceptual design (deterministic versus stochastic, long-term memory versus no long-term memory) and not by the underlying search method.

The main idea behind the two approaches is to use a pool of solutions as long-term memory that stores each unique solution found during the search process (Line 17/19 in Algorithm 8/9). At every point in time, the algorithms use one of these solutions (*x*) as their current incumbent solution (Line 13/14 in Algorithm 8/9). At the beginning of each iteration, all solutions in the pool are scheduled according to the currently known real travel speeds (Line 4/4 in Algorithm 8/9). To guarantee the feasibility of all solutions in the pool, solutions that are incompatible with decisions made in the current incumbent solution get eliminated from the pool when necessary (Line 7/7 in Algorithm 8/9). The resulting solution is thus defined by the sequence of actions taken during execution. Then all newly known requests are inserted into every solution in the pool.

The main difference between MPA and MSA is that the latter incorporates stochastic information in the search process, while the former does not. For this purpose, the multiple scenario approach uses sampled future travel speed deviations in a similar way as dynamic S-VNS does. Contrary to dynamic S-VNS, it does not use the set of dynamically selected sampled accidents to compare a candidate solution to the current incumbent solution but rather to select the current incumbent solution out of all solutions in the pool. Therefore, each solution has to be rescheduled using the information about each of the used samples; then the resulting average solution qualities are compared. As for S-VNS, the size of the used set of samples $\overset{‾}{Z}$ is dynamically adjusted, such that the execution time of one evaluation equals 1 second (Line 15 in Algorithm 9).

According to Bent and Bent and Van Hentenryck (2004), the best strategy to select the current incumbent solution uses a consensus function, similar to a least commitment strategy (i.e., select the solution most similar to all other solutions in long-term memory). Previous findings for the DSDARP (Schilde et al., 2011) indicate that this strategy may not be the best choice in all circumstances though. Because the dynamic DARP with stochastic, time-dependent travel speeds is structurally similar to the DSDARP, we decided to use the best solution in the pool as our current incumbent solution.

## 5 Numerical analysis

### 5.1 Test instances

The ride times in our test instances are based on historical floating car data gathered during a project in the city of Vienna in 2009. Descriptions of the calculations of time-dependent travel times based on FCD-data are available in Reinthaler, Nowotny, Weichenmeier, and Hildebrandt (2007), Laborczi, Linauer, and Nowotny (2006), and Linauer and Nowotny (2006), and the instances can be downloaded from http://www.jku.at/plm/instances.

For this study we assume that a day consists of 24 time intervals, each of one hour in length. For each link inside the used real-world road network and each of the intervals, we can determine an average vehicle speed. These travel speeds serve as the planning basis for the deterministic approaches (see Section 4). In addition, we generate stochastic deviations from these average velocities using the congestion circle procedure described in Section 3.1.

We create our transportation requests on the basis of distribution parameters derived from real-world data on the daily operations of an Austrian ambulance service provider during the course of one year. A detailed description of the process for determining the distribution parameters can be found in Schilde et al. (2011). In addition, we used a set of geographic patient locations and hospital sites in the city of Vienna, corresponding to the original locations serviced during the year. To model the geographic distribution of the generated requests, the set includes an occurrence counter for each location, which then can assign locations to created requests via roulette wheel. By sampling the distribution of the interarrival time of two consecutive requests, we determine the arrival times for all requests. Similarly, the distribution of the time between an incoming request and the latest time of arrival at the hospital is sampled, to construct the requests’ time windows. By varying the distribution of interarrival times, we create instances with N={215,430,578,762} requests arising during a ten-hour work day. In what follows, we refer to these instance sizes as “tiny”, “small”, “medium”, and “large”. Each instance consist of 50% inbound (home to hospital) and 50% outbound (hospital to home) requests.

For each of the four instance sizes, we create eight instances with different percentages of dynamic requests. Thus the instances were created such that the requests can be selected as static or dynamic by defining the desired degree of dynamism. For our computational experiments, we use degrees of dynamism, Λ={0%,15%,30%,45%,60%,75%,90%,100%}, to determine how this factor influences our results.

Finally, we calculate a greedy solution for each test instance using the modified cheapest insertion procedure described in Section 4.3, under the assumption that all requests are known a priori. The number of vehicles used in this solution is increased by 10%, which provides the maximum number of vehicles available to all four solution algorithms.

### 5.2 Numerical results

Computational testing is performed using non-parallel C++ implementations of the described algorithms. Compilation is done using the GNU C++ compiler in its version 4.3 on SuSE Linux Enterprise Server 11. All calculations are performed using one core of an Intel Xeon E7-8837 (WestmereEX, 2.66 GHz) and 8 GB of memory.

In what follows, we present the results obtained in terms of relative solution quality. The results for the stochastic methods therefore are given as a percentage gap from the results obtained by their deterministic counterparts. A positive percentage always indicates that the corresponding method yields better solution quality than the compared approach, whereas a negative value indicates the opposite. Using 10 independent runs, we also calculate the 95% confidence intervals for the differences between each pair of objective function values. We compare the results obtained by our two deterministic methods (dynamic VNS and MPA) using time-dependent travel speeds against results obtained by the same methods using constant average travel speeds. These two time-independent versions are denoted AVNS and AMPA in the following discussion. These results are also presented as the relative gap from those obtained when using time-dependent travel speeds.

As we mentioned previously, the main focus of our research was to determine if our stochastic algorithms (dynamic S-VNS and MSA) obtain better solutions for this problem than deterministic methods (dynamic VNS and MPA). We provide summaries of the results obtained by dynamic S-VNS and MSA depending on Λ in Figs. 6 and 7, respectively, though we do not report the results for Λ=0%, because they are strongly negative and would distort the scale. A complete list of all results appears in Table 1 for the stochastic methods when dynamic requests are present, Table 2 for the stochastic methods without any dynamic requests, and Table 3 for the deterministic methods with time-independent travel speeds (all relative to the results obtained by the deterministic methods with time-dependent travel speeds).

Not all stochastic solution approaches appear equally suitable for the problem at hand. Whereas dynamic S-VNS is a promising concept, MSA does not obtain the same solution quality, even with the same underlying search procedure (see Fig. 5). Especially in larger problem settings with few dynamic requests, MSA cannot compete with the results obtained by MPA (see Fig. 7). The quality of the results obtained by the latter is very similar to that of the results obtained by dynamic VNS though. Nevertheless, MSA already offers a powerful approach for different problem settings (e.g., the vehicle routing problem with stochastic customers).

The reason for the relative disadvantage of MSA compared with dynamic S-VNS reflects the computational demands of evaluating a pool of solutions based on sampled accidents, which is far greater than the demand associated with comparing a single candidate solution against the current incumbent. Therefore, the size of the used sample set is much smaller for MSA than for dynamic S-VNS (e.g., 1.04 vs. 142.80 samples on average; maximum of 42 vs. 161 samples for the small instance during one run with an average of 682.09 plans in the pool of MSA).

**Fig. 4:**
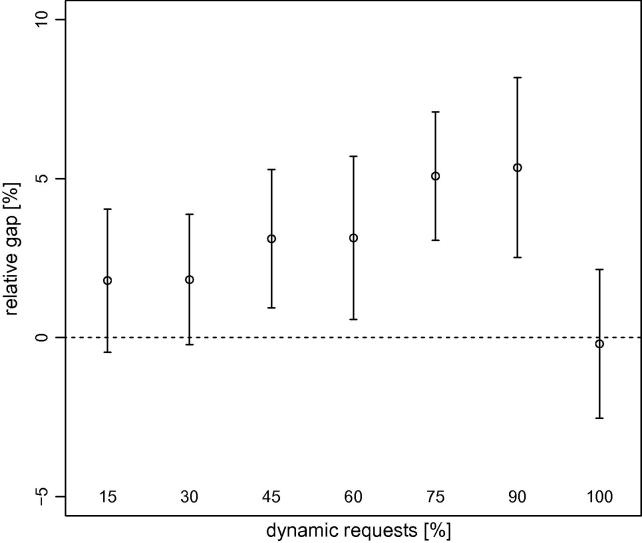
Average (circles) and 95% confidence intervals (whiskers) of the gaps between the primary objective function values obtained by dynamic VNS and those obtained by dynamic S-VNS, depending on the percentage of dynamic requests aggregated over all instance sizes. Positive values indicate superior results obtained by the latter.

**Fig. 5:**
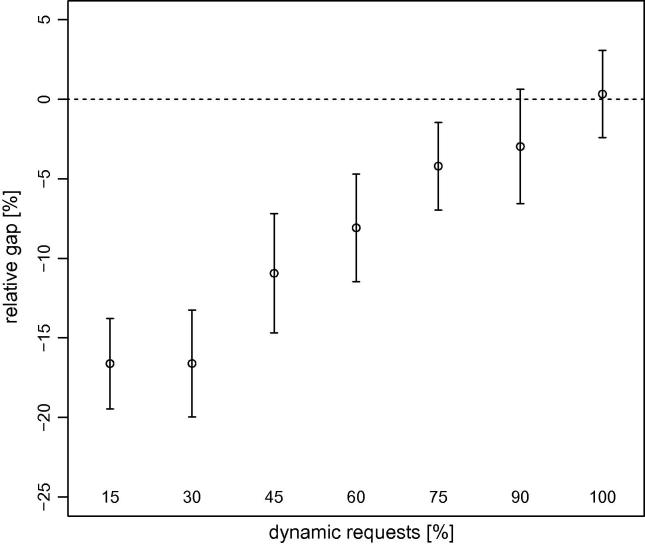
Average (circles) and 95% confidence intervals (whiskers) of the gaps between the primary objective function values obtained by MPA and those obtained by MSA, depending on the percentage of dynamic requests aggregated over all instance sizes. Positive values indicate superior results obtained by the latter.

**Fig. 6:**
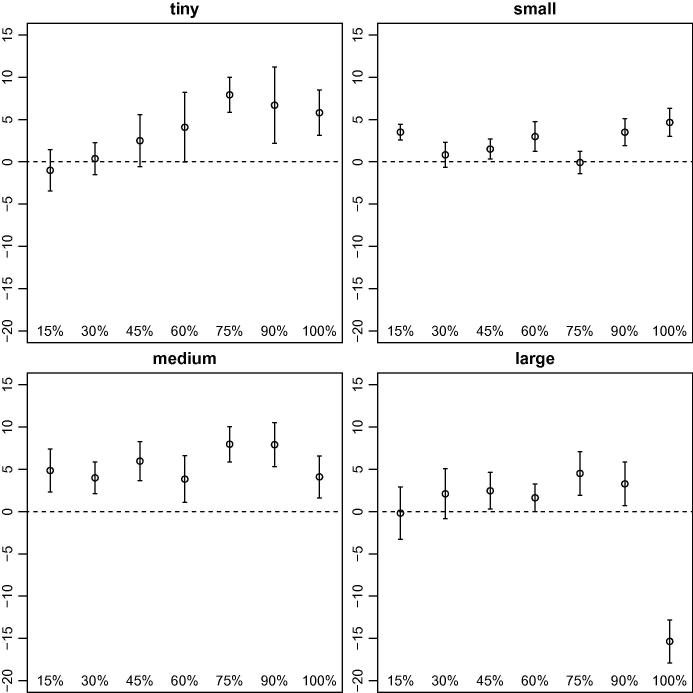
Average (circles) and 95% confidence intervals (whiskers) of the gaps between the primary objective function values obtained by dynamic VNS and those obtained by dynamic S-VNS, depending on the percentage of dynamic requests grouped by instance size. Positive values indicate superior results obtained by the latter.

**Fig. 7:**
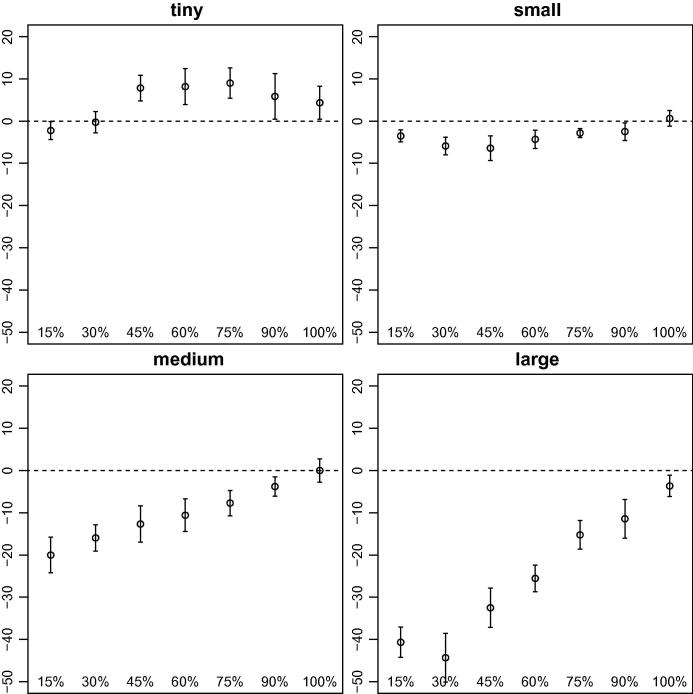
Average (circles) and 95% confidence intervals (whiskers) of the gaps between the primary objective function values obtained by MPA and those obtained by MSA, depending on the percentage of dynamic requests grouped by instance size. Positive values indicate superior results obtained by the latter.

Our findings also clearly show that incorporating stochastic information about future traffic accidents while planning routes for the dynamic DARP does not automatically guarantee better results. In certain conditions, the additional effort put into stochastic solution methods can result in superior solutions, largely depending on the amount of dynamism inherent in the problem. It appears that dynamic S-VNS works best in settings with 45% to 90% of dynamic requests (see Fig. 4). For fully static instances and very large fully dynamic instances, deterministic dynamic VNS clearly is the method of choice. For instances with relatively few dynamic requests, neither of the two methods dominates.

The competitiveness of deterministic methods in scenarios without dynamic requests can be explained by the extended periods of time available for algorithms to react to changes in travel speeds. Every time a real travel speed is revealed to the method, the solution can be fully reoptimized before the next vehicle departs for its next stop. In situations with dynamically arising requests, the time available for recalculation is rather limited, because the new request must be addressed, which requires more robust solutions in the first place. For instances with many dynamic requests, the opposite effect seems to be true. That is, the time for reoptimization is very limited, but a deterministic method can make more out of it, because it is significantly faster as a result of the conceptual overhead introduced by stochastic methods such as dynamic S-VNS.

Using time-dependent travel speeds in deterministic methods outperforms constant travel speeds, as expected (see Table 3). The additional possible improvements obtained from the use of stochastic travel speeds is even greater than expected. The mean expected deviation from average time-dependent travel speeds due to accident influences is only 1.02% (see Section 3.1), so obtained improvements of up to 8.99% may seem surprising at a first glance. The explanation for this effect is quite simple though. Let the planned travel time between two stops along a route be T^(t,d)=100 minutes. Then an increase of 1 minute ($T_{\mathrm{real}}=101$ minutes) might cause an increase in the primary objective by 1 minute as well, but only if the remainder of the route can compensate for this 1 minute shift. Otherwise, all further stops along the route might be shifted by 1 minute too (or by more or less, due to the interval boundaries), causing an increase in the primary objective of up to 1 minute times the number of further stops along the route. That is, a very small unexpected change in travel speeds can cause large changes in solution quality.

## 6 Summary and outlook

With this article we present adapted versions of two conceptually similar pairs of metaheuristic solution approaches for the dynamic DARP with stochastic, time-dependent travel speeds. The first pair consists of a dynamic variable neighborhood search method (dynamic VNS) and a stochastic variant thereof (dynamic S-VNS). The second pair includes the multiple plan approach (MPA) and the multiple scenario approach (MSA). All four methods use our implementation of dynamic VNS as a search component. Our main aim is to determine if stochastic algorithms that take information about future traffic accidents into account lead to better solutions than deterministic methods using only average, time-dependent travel speeds.

We test all algorithms on sets of real-world inspired test instances. Our findings show that dynamic S-VNS works well for settings with 45% to 90% dynamic requests and between around 400 and 600 requests in total. In purely static or very dynamic settings, the deterministic dynamic VNS leads to better results. For the problem at hand, the additional effort of managing a pool of found solutions does not offer additional benefits. The concept of maintaining a pool of solutions in MSA turns out to be unsuitable for this problem setting, except for very small instances and medium degrees of dynamism.

In summary it can be stated that the exploitation of historical accident information within our dynamic S-VNS for the dynamic DARP with stochastic, time-dependent travel speeds leads to significantly better results in cases with 45% to 90% of initially unknown requests.

In further work, we plan to study a combination of this problem with expected return transports to determine if more information about future stochastic influences might be even more beneficial to solution quality or offer additional insights. Including approaches for dynamic re-calculation of the underlying shortest paths based on time-dependent travel speeds could also be an interesting extension to the work presented in this article. An extension of the problem at hand with heterogeneous passengers, a heterogeneous vehicle fleet, and/or multiple depots could be of interest as well. Finally, additional real-world aspects, such as driver-related constraints, might be informative.

## Figures and Tables

**Table 1:** Average solution quality, depending on instance size and share of dynamic requests Λ. All results are relative to those obtained with the corresponding deterministic solution method (VNS – S-VNS and MPA – MSA). Positive values (highlighted in bold) indicate superior results obtained by the stochastic method mentioned in the column header. Dissatisfaction refers to the primary objective, calculated as the sum of tardiness, earliness, and ride time violation; *#Vehicles* is the secondary objective, calculated as the number of vehicles used; and *Duration* is tertiary objective, equal to the total route duration.

Size	Λ (%)	Dissatisfaction (%)		*#Vehicles (%)*		*Duration (%)*	
		S-VNS	MSA	*S-VNS*	*MSA*	*S-VNS*	*MSA*
Tiny	15	−1.00	−2.24	−*15.97*	−*15.29*	−*12.69*	−*11.46*
Tiny	30	**0.37**	−0.26	−*12.97*	−*20.08*	−*10.88*	−*18.01*
Tiny	45	**2.50**	**7.83**	−*8.23*	−*19.31*	−*11.97*	−*13.50*
Tiny	60	**4.09**	**8.16**	−*12.92*	−*12.77*	−*14.15*	−*12.81*
Tiny	75	**7.92**	**8.99**	−*8.94*	−*21.40*	−*15.17*	−*11.49*
Tiny	90	**6.70**	**5.84**	−*17.65*	−*20.18*	−*17.14*	−*9.89*
Tiny	100	**5.81**	**4.34**	−*20.09*	−*20.09*	−*21.18*	−*12.73*
Small	15	**3.51**	−3.53	−*9.15*	−*12.22*	−*7.53*	−*12.79*
Small	30	**0.82**	−5.89	−*5.75*	−*12.16*	−*7.46*	−*12.43*
Small	45	**1.50**	−6.41	−*7.04*	−*12.16*	−*9.77*	−*14.83*
Small	60	**2.99**	−4.33	−*10.38*	−*13.41*	−*11.48*	−*13.17*
Small	75	−0.08	−2.82	−*6.31*	−*13.83*	−*10.82*	−*13.46*
Small	90	**3.50**	−2.48	−*9.93*	−*10.77*	−*13.93*	−*12.12*
Small	100	**4.66**	**0.62**	−*12.20*	−*12.05*	−*16.97*	−*13.25*
Medium	15	**4.85**	−20.02	−*3.33*	−*9.09*	−*8.94*	−*13.15*
Medium	30	**3.99**	−15.95	−*4.49*	−*8.42*	−*10.51*	−*13.87*
Medium	45	**5.97**	−12.67	−*5.11*	−*8.15*	−*10.21*	−*13.31*
Medium	60	**3.84**	−10.60	−*3.99*	−*10.82*	−*12.66*	−*12.11*
Medium	75	**7.96**	−7.72	−*5.01*	−*9.82*	−*13.16*	−*13.16*
Medium	90	**7.91**	−3.80	−*11.83*	−*11.47*	−*17.78*	−*13.56*
Medium	100	**4.10**	0.00	−*12.67*	−*12.30*	−*19.38*	−*13.44*
Large	15	−0.18	−40.67	−*5.45*	−*10.51*	−*13.22*	−*15.81*
Large	30	**2.11**	−44.34	−*4.52*	−*9.47*	−*12.79*	−*16.19*
Large	45	**2.46**	−32.50	−*4.45*	−*9.41*	−*13.78*	−*14.05*
Large	60	**1.63**	−25.55	−*5.83*	−*13.12*	−*13.49*	−*13.88*
Large	75	**4.52**	−15.25	−*6.08*	−*11.92*	−*14.39*	−*13.81*
Large	90	**3.28**	−11.45	−*4.74*	−*8.80*	−*15.98*	−*13.71*
Large	100	−15.36	−3.66	−*14.74*	−*10.21*	−*12.68*	−*13.16*

Avg.		**2.87**	−8.44	−*8.92*	−*12.83*	−*13.22*	−*13.40*

**Table 2:** Average solution quality, depending on instance size with no dynamic requests (Λ=0%). All results are relative to those obtained with the corresponding deterministic solution method (VNS – S-VNS and MPA – MSA). Negative values indicate inferior results obtained by the stochastic method mentioned in the column header. Dissatisfaction refers to the primary objective, calculated as the sum of tardiness, earliness, and ride time violation; *#Vehicles* is the secondary objective, calculated as the number of vehicles used; and *Duration* is tertiary objective, equal to the total route duration.

Size	Λ (%)	Dissatisfaction (%)		*#Vehicles (%)*		*Duration (%)*	
		S-VNS	MSA	*S-VNS*	*MSA*	*S-VNS*	*MSA*
Tiny	0	−4.89	−4.28	−*17.41*	−*22.66*	−*4.17*	−*14.82*
Small	0	−4.65	−5.21	−*11.14*	−*12.97*	−*0.34*	−*12.68*
Medium	0	−55.97	−20.91	*0.00*	−*9.36*	−*1.02*	−*13.90*
Large	0	−115.17	−44.18	*1.63*	−*7.95*	−*2.82*	−*12.48*

Avg.		−45.17	−18.65	−*6.73*	−*13.24*	−*2.09*	−*13.47*

**Table 3:** Average solution quality, depending on instance size and share of dynamic requests Λ, using deterministic solution methods with average (time-independent) travel speeds. All results are relative to those obtained by the corresponding solution method using time-dependent travel speeds (VNS – AVNS, MPA – AMPA). Negative values indicate superior results obtained by the method with time-dependent travel speeds (i.e., it is always superior). Dissatisfaction refers to the primary objective, calculated as the sum of tardiness, earliness, and ride time violation; *#Vehicles* is the secondary objective, calculated as the number of vehicles used; and *Duration* is tertiary objective, equal to the total route duration.

Size	Λ (%)	Dissatisfaction (%)		*#Vehicles (%)*		*Duration (%)*	
		AVNS	AMPA	*AVNS*	*AMPA*	*AVNS*	*AMPA*
Tiny	0	−54.81	−54.75	*4.45*	−*22.66*	*8.07*	*10.75*
Tiny	15	−52.88	−48.91	−*2.10*	−*15.29*	*5.98*	*8.73*
Tiny	30	−56.33	−49.74	−*3.77*	−*20.08*	*3.34*	*6.55*
Tiny	45	−55.38	−45.79	*2.88*	−*19.31*	*1.80*	*4.84*
Tiny	60	−52.99	−43.07	−*0.83*	−*12.77*	*4.19*	*4.60*
Tiny	75	−44.48	−40.70	*2.44*	−*21.40*	*3.27*	*4.46*
Tiny	90	−46.34	−37.35	−*1.68*	−*20.18*	*3.37*	*3.96*
Tiny	100	−49.37	−43.02	−*2.62*	−*20.09*	−*0.45*	*1.92*
Small	0	−65.73	−63.85	−*0.23*	−*12.97*	*4.75*	*6.14*
Small	15	−60.05	−59.17	*1.60*	−*12.22*	*5.34*	*7.62*
Small	30	−63.81	−62.70	−*0.23*	−*12.16*	*4.17*	*5.61*
Small	45	−62.39	−62.85	−*3.76*	−*12.16*	*2.03*	*3.80*
Small	60	−51.89	−52.60	*0.00*	−*13.41*	*2.24*	*4.51*
Small	75	−55.72	−55.32	−*0.23*	−*13.83*	*0.77*	*1.87*
Small	90	−41.73	−44.58	*0.00*	−*10.77*	*1.03*	*2.26*
Small	100	−35.79	−39.62	−*0.96*	−*12.05*	*2.02*	*1.40*
Medium	0	−53.43	−47.46	*3.61*	−*9.36*	*4.50*	*6.19*
Medium	15	−50.98	−47.12	*2.22*	−*9.09*	*4.04*	*6.59*
Medium	30	−52.43	−45.65	*0.96*	−*8.42*	*5.02*	*6.33*
Medium	45	−45.59	−44.71	*3.04*	−*8.15*	*5.63*	*5.34*
Medium	60	−47.05	−45.24	*3.35*	−*10.82*	*3.13*	*4.67*
Medium	75	−45.78	−48.10	*2.10*	−*9.82*	*2.11*	*2.64*
Medium	90	−46.88	−46.11	*1.83*	−*11.47*	*1.70*	*1.69*
Medium	100	−39.54	−42.39	−*1.56*	−*12.30*	*2.04*	*0.46*
Large	0	−65.85	−63.50	*1.17*	−*7.95*	*3.73*	*6.68*
Large	15	−71.60	−74.48	*2.20*	−*10.51*	*2.62*	*3.87*
Large	30	−64.58	−70.47	*0.00*	−*9.47*	*3.10*	*3.94*
Large	45	−69.26	−67.33	*1.05*	−*9.41*	*1.16*	*3.18*
Large	60	−67.43	−65.43	*0.48*	−*13.12*	*1.46*	*4.42*
Large	75	−64.18	−64.08	−*1.34*	−*11.92*	*1.12*	*1.96*
Large	90	−63.47	−65.43	−*0.36*	−*8.80*	*1.34*	*0.66*
Large	100	−62.04	−62.69	−*2.64*	−*10.21*	*1.13*	*1.43*

Avg.		−66.05	−66.68	*0.07*	−*10.18*	*1.96*	*3.27*
